# Plant growth promotion effect of plasma activated water on *Lactuca sativa* L. cultivated in two different volumes of substrate

**DOI:** 10.1038/s41598-020-77355-w

**Published:** 2020-12-01

**Authors:** Vasile Stoleru, Radu Burlica, Gabriela Mihalache, Delicia Dirlau, Silvica Padureanu, Gabriel-Ciprian Teliban, Dragos Astanei, Alexandru Cojocaru, Oana Beniuga, Antoanela Patras

**Affiliations:** 1 “Ion Ionescu de la Brad” University of Agricultural Sciences and Veterinary Medicine, 3 M. Sadoveanu Alley, 700490 Iaşi, Romania; 2 “Gheorghe Asachi” Technical University of Iaşi, 67 Bd. D. Mangeron, 700050 Iaşi, Romania; 3 Integrated Center of Environmental Science Studies in the North East Region (CERNESIM), “Alexandru Ioan Cuza” University of Iasi, 11 Carol I, 700506 Iaşi, Romania

**Keywords:** Plant sciences, Engineering

## Abstract

Plasma activated water (PAW) can represent an alternative to chemical fertilizers in agriculture. The effects of PAW treatment applied in two concentrations (1.5 or 3.0 mg L^−1^ NO_3_^−^) on some morphological, physiological, biochemical parameters and yield of *Lactuca sativa* L. grown in two different pot volumes (400 or 3200 cm^3^) were investigated in this study. The results showed that both PAW concentrations did not influence the germination, once the process was initiated. Positive effects of the treatments were registered on the length of radicle and hypocotyls of lettuce at a concentration of 1.5 mg L^−1^ NO_3_^−^ (PAW I), the chlorophyll content was significantly increased at a concentration of 3.0 mg L^−1^ NO_3_^−^ (PAW II) and bigger pot volume, also the foliar weight and area. No significant differences between the treated and untreated plants were recorded for the root weight, leaf length and width. The dry weight was significantly higher for the lettuce treated with PAW I and II grown in big volume pots at 57 days after transplanting (DAT) and small volume pots at 64 DAT. The nitrites content of the lettuce grown in big pots was lower than of the lettuce grown in small pots, regardless of the PAW treatment. Contrary, the nitrates content was higher in the lettuce grown in big pots (up to 36.4 mg KNO_3_/g DW), compared to small pots (under 0.3 mg KNO_3_/g DW).

## Introduction

In the last two decades, as a result of the constant population growth in the world, horticultural systems for the production of vegetables in greenhouses on artificial substrates (soilless crop) have been technologically improved by introducing measures aimed to increase production. Only in the last year, the demographics of the world’s population grew by 4.5 million people^1^. Hydroponic system is a method of growing plants in which the nutrients can be artificially efficiently supplied to crops in their mineral form. This method has many advantages, including reduction in pest problems, constant feeding of the roots with nutrients and high productivity^2^.

At the same time, for food safety, the fresh products obtained on artificial substrates must be both, nutritious and free of contaminants, regardless of the inputs used.

High yield of agricultural crops relies on planting high vigor seeds. In order to achieve high yields, treatments for the protection of seeds against pathogens and pests and for stimulation of germination and fertilization are widely used^3^.

Recently, the non-thermal plasma (NTP) technology has focused its attention on the field of agriculture as an alternative to chemical biostimulators. Physical plasma is considered to be the fourth state of matter and it constitutes the major part of the visible Universe^4–6^.

The use of cold plasma is better known in agricultural systems for controlling microorganisms’ development in storage areas^7–9^ or for a safe packaging of foodstuffs^10^, to determine the reduction of pesticides in fruit and vegetables^11,12^, for hygienic decontamination^5,13^ or drought stress^14^.

Cold plasma technologies are already considered as an alternative to other consecrated treatment methods for air, water, soil, seeds, sprouts, seedlings or plants, meant to improve their characteristics. Studies on the properties of water treated with cold plasma highlighted the organic contaminants removal^15–17^, and the generation of reactive species like H_2_O_2_, (reactions 1–4)^18,19^, respectively NO_3_^−^ and NO_2_^–^^20^.

Nitrogen oxides can form nitrites in liquid phase which react with hydrogen peroxide to form nitrate (reactions 7). The nitrates affect the conductivity and pH through the formation of acids and ions in water and reduce the formation of hydrogen in gas phase and hydrogen peroxide in water droplets, (reactions 5–9). Ionization by electron/ion impact may occur by the reactions:

1 $${\text{H}}_{{2}} {\text{O}} + {\text{e}}^{ - } \to {\text{H}}^{ + } + {\text{OH}}* + {\text{2e}}^{ - }$$

2 $${\text{2H}}_{{2}} {\text{O}} + {\text{e}}^{ - } \to {\text{H}}_{{3}} {\text{O}}^{ + } + {\text{OH}}^{ - } + {\text{e}}^{ - }$$

H_2_O_2_ may result through an overall reaction such as:

3 $${\text{2H}}_{{2}} {\text{O}} \to {\text{H}}_{{2}} {\text{O}}_{{2}} + {\text{H}}_{{2}}$$

H_2_ and O_2_ may also be formed through an overall reaction such as:

4 $${\text{H}}_{{2}} {\text{O}} \to {\text{H}}_{{2}} + \raise.5ex\hbox{$\scriptstyle 1$}\kern-.1em/ \kern-.15em\lower.25ex\hbox{$\scriptstyle 2$} {\text{ O}}_{{2}}$$

In the case of air or nitrogen as carrier gas in the process of plasma activated water (PAW) generation, the formation of nitrogen oxides occurs by the reactions:

5 $${\text{N}}_{{2}} + {\text{e}}^{ - } \to {\text{2N}} + {\text{e}}^{ - }$$

6 $${\text{O}}_{{2}} + {\text{e}}^{ - } \to {\text{2O}} + {\text{e}}^{ - }$$

7 $${\text{N}} + {\text{O}} \to {\text{NO}}$$

8 $${\text{NO}} + {\text{O}} \to {\text{NO}}_{{2}}$$

The nitrogen oxides affect the pH and the conductivity of the water through the formation of ions and acids as described by the reaction^21^:

9 $${\text{NO}}_{{2}} + {\text{OH}} \to {\text{HNO}}_{{3}}$$

Plasma activated water is obtained from tap water, distillated water or demineralized water exposed to the action of a plasma-generating electrical discharge in vacuum, air, inert gases or other, with different reactor configurations^22^.

From a physiological standpoint, the use of plasma in agriculture is better known for the positive effects on seed germination^7,23,24^ or on early growth plant^21,25,26^. For instance, NTP treatment (140 W–160 W power) on maize seed increased the germination rates by 28%, the length of the wheat radicle was effectively increased by 8.7 cm and 3.3 cm, the dry weight of the wheat was increased by 10.1%; and germinative energy of the aging seeds have been greatly increased^27^.

Also, researches on the use of NTP have shown its potential for increasing the microbiological safety of vegetables and dried fruits^8,9^.

Cold plasma treatment (CPT) can inactivate microorganisms contaminating food products without a marked temperature increase^28^. The cold plasma contains energetic reactive species, such as ultraviolet (UV) photons, electrons, positive and negative ions, free radicals and excited or non-excited molecules and atoms^29^.

The consumption of leafy green vegetables (including herbs) has increased over the last 20 years in the European market, at an annual growth rate of about 4%; this is the reason why this food category is recognized as one of the most profitable in the fruit and vegetables segment. As the result of an upward trend observed during the last decade, lettuce is cultivated on a total area of over 1.2 mil. ha worldwide, with a global production of approximately 27 mil t^30^.

Lettuce (*Lactuca sativa* L.) is one of the most consumed fresh vegetables in the world. It can be grown on soil or hydroponic conditions^31^. The common practice used to improve the yield is applying chemicals, but these are known to affect the environment^32^.

In protected areas, lettuce is technologically grown on artificial substrates, which are often poor in nutrients^33^ and need to be supplied by organic or chemical nutrients, under high food safety conditions, without high nitrate content, or by unconventional sources as non-thermal plasma^22^.

In general, the NTP treatments applied to seeds do not have a significant influence on the yield, from both quantitatively and qualitatively points of view. Good results were obtained for the percentage of sprouted seeds, the length of radicles and hypocotyls by applying NTP to the *Asteraceae* family^34^.

Inorganic nitrates and nitrites are naturally occurring compounds in vegetables. A wide range of nitrates content in vegetables from 1 to more than 1000 mg/100 g has been reported^35^ and lettuce hold one of the top positions concerning the high content. Especially fenugreek, tarragon and lettuce, but also spinach and carrot, possess a strong capacity to accumulate nitrates^36^. Bahadoran et al*.*^35^ studied the nitrates and nitrites contents in 13 leafy vegetables and found that lettuce had a very high level (365 ± 232 mg/100 g) after fenugreek, and tarragon. Excepting the cultivar and the agricultural practices, the climate and in particular the light conditions are the main determinant factors for the nitrates content in lettuce. Nitrate accumulation in lettuce is greater when light intensity is low because there is less energy to convert it into reduced forms of nitrogen^37^. The amount of nitrate existing in plant at a certain moment is the result of the balance between the amount absorbed and that used in protein genesis^38^. According to the European Commission Regulations, the maximum level for «lettuce grown under cover» is 4000 mg NO_3_^−^/kg^39^.

The aim of the present study was to assess, in dynamics, the influence of plasma activated water (PAW) treatment on the Shangore F1 lettuce. Several parameters were followed during the experiments, as the rate of seed germination, the length of hypocotyl and radicle (in the germination process), the surface area, the length and width of the leaves, the foliar and radicular weight, the content of chlorophyll pigments (in the growth process) and the content of nitrites and nitrates (at the harvest time).

## Results and discussion

### Germination and seedling growth under PAW treatment

**Figure 1:**
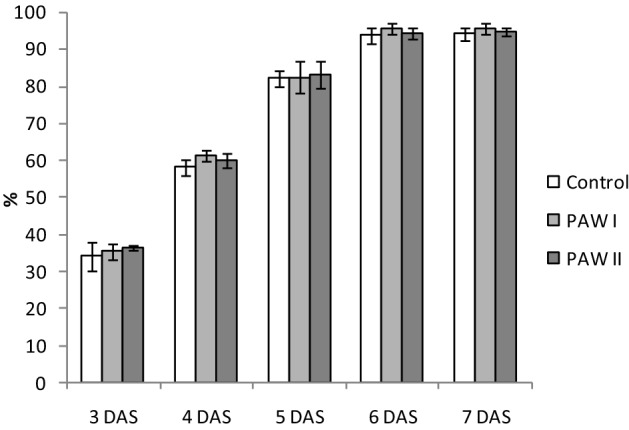
The dynamic of the germination rate of lettuce seeds for *P* < 0.05 in the absence (control) or presence of PAW I (1.5 mg L^−1^ NO_3_^−^) or PAW II (3.0 mg L^−1^ NO_3_^−^). Results are means ± SD (n = 3). *DAS* days after sowing.

The results of the PAW’s effect on the lettuce seed germination are shown in Fig. 1. At 7 days after sowing (DAS), the seed germination rate was between 94% (Control) and 95% (PAW I and PAW II). The plasma treatment did not determine significant differences in the germination rates of the seeds; the differences between treated and untreated seeds were less than 1%. Previous researches on lettuce reported similar results when PAW was used at concentrations of 34 and 54 mg L^−1^ NO_3_^40,41^. Also, by applying dielectric barrier discharge (DBD) to the tomato seeds similar results were obtained, suggesting that the treatment did not produce damages to the seeds’ cell membrane^3^, stimulating the germination process^42^. The effect of PAW on germination depends on many factors such as plant species, time of application, type of discharge, type of plasma treatment, type of water, experimental conditions^43^. For instance, Puac et al*.*, in a study regarding *P. tomentosa* stated that pH values of the PAW treatments are very important in the germination of the seeds and the growth of the seedling: very high or very low pH affects the enzymes activity^44^. In our study, the differences between the pH of the treatments and the pH of control were very small, avoiding the possible deleterious effects that could appear in the case of a significant change of pH. The pH of PAW I and II were 6.54 and 5.88 respectively, being within the optimal pH limits for the lettuce growth (5.8–6.5)^45^. Regarding the pH of the distilled water used for the control group, its value was 6.9. Therefore, the pH values of the treatment did not influence the germination process. Zhang et al.^46^, registered an increase of 50% in the germination of lentil seeds treated with PAW produced by atmospheric pressure He plasma jet and applied at the moment of seed incubation. In our study, the application of PAW at 3 DAS highlighted that once the germination process was initiated, the treatments did not produce significant modifications compared to the control, regardless of concentration used and number of days after sowing, the differences between the groups varied between 1 and 2%.

Usually, during the normal germination process of the seeds, after the membrane breaks, the radicle immediately appears. At that moment, the germination process is not considered to be finished, because the hypocotyls must also appear, in order to grow a new plant^40^.

**Figure 2:**
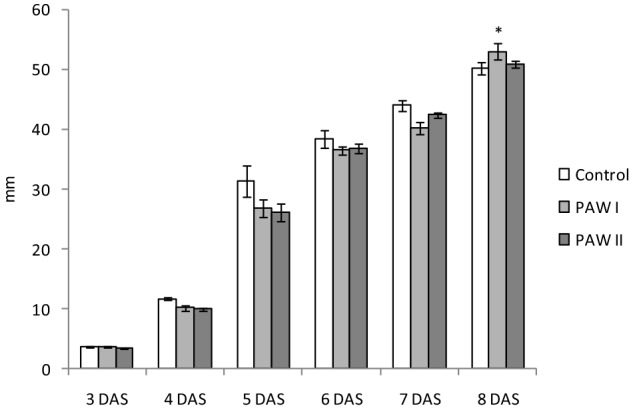
The dynamic of radicle growth in the absence (control) or presence of PAW I (1.5 mg L^−1^ NO_3_^−^) or PAW II (3.0 mg L^−1^ NO_3_^−^). Results are means ± SD (n = 3). Asterisk means a significant difference (*P < 0.05) vs. control. *DAS* days after sowing.

The data shown in Fig. 2, regarding the measurements of the radicle length, indicate that at 3 and 4 DAS there are no significant differences between the treated and untreated seeds. At 8 DAS a significant better result was recorded in case of PAW I treatment, with an average radicle length of 53 mm compared to 50.9 mm for PAW II and 50.2 mm for the control. This suggests that the treatment with 1.5 mg L^−1^ NO_3_^−^ is favorable for the process of radicle growth 8 days after sowing.

**Figure 3:**
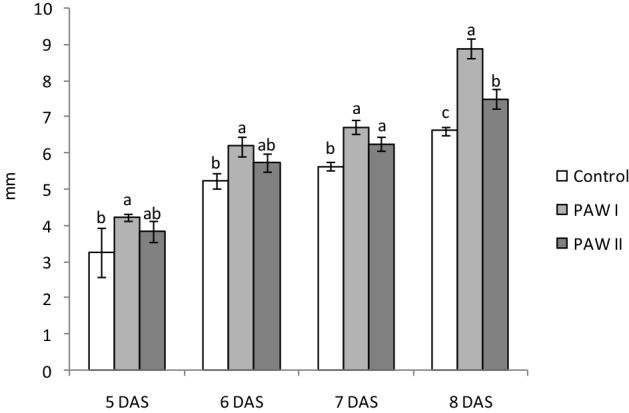
Dynamic of lettuce hypocotyls growth in the absence (control) or presence of PAW I (1.5 mg L^−1^ NO_3_^−^) or PAW II (3.0 mg L^−1^ NO_3_^−^). Results are means ± SD (n = 3). Different letters mean significant differences between the groups, according to Tukey posthoc test (*P* < 0.05). *DAS* days after sowing.

The hypocotyls dynamic showed that between 5 and 8 DAS the PAWs effect was visible (Fig. 3). Thus, significant better results compared to the control (e.g. 8.9 mm vs. 6.6 mm at 8 DAS) were obtained at a content of 1.5 mg L^−1^ NO_3_^−^ (PAW I), which for the subsequent process of growth and development of the plant is a positive aspect, determining a higher force and implicitly increased production. Significant differences were also observed between PAW II treatment and control starting with 7 DAS, but the increases were less important compared to PAW I.

Similar results were recorded by Fan et al*.*^47^ in a study regarding mung bean, where the positive effects on the seedlings growth decreased along with the increased discharge time. The lower positive effects of PAW II on the radicle and hypocotyls growth of lettuce seedlings can be due to the higher concentrations of reactive species (3.0 mg L^−1^ NO_3_^−^ and 1.65 mg L^−1^ H_2_O_2_) that might have had a detrimental effect, slowing the growth processes. It is well known that at low concentrations, nitric oxide (NO) and reactive oxygen (ROS) have positive effects on the growth and development of plants. However, at high concentrations, reactive species can become toxic, inhibiting or slowing different developmental processes^47^. Another reason which could have contributed to the lesser increase of the lettuce seedlings treated with PAW II might be the pH of the treatment (5.88) which is at the inferior limit of the optimal pH needed for the growth of lettuce^45^.

Positive effects of PAW treatment were also recorded in 2011 for rye. After treating the rye seeds for 5 min with plasma activated water, the length of the roots was double while the length of the germ coleoptiles was 1.5 times higher compared to that of the untreated control^48^.

In conclusion, PAW treatment did not affect the seed germination of lettuce once the process had started, but had a stimulatory effect on the growth of radicle and hypocotyls, especially at a concentration of 1.5 mg L^−1^ NO_3_^−^, the increases being of 5.58%, respectively 34.85% compared to the untreated control.

### Influence of PAW on total chlorophyll content

The content of photosynthetic pigments is usually correlated with the abiotic factors, as different treatments, and the phenophase of the plant. In our research, the effect of PAW treatments on the chlorophyll content of lettuce plants was analyzed in dynamic at 50, 57 and 64 days after transplanting (DAT).

**Figure 4:**
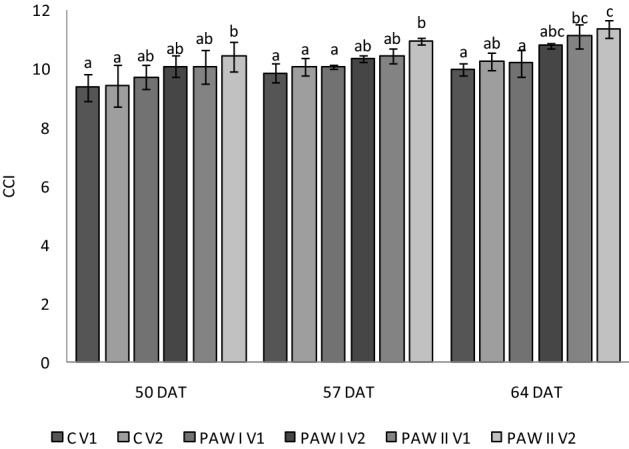
Dynamic of chlorophyll content of lettuce leaves at 50, 57 and 64 DAT under different treatments (C = control, PAW I = 1.5 mg L^−1^ NO_3_^−^; PAW II = 3.0 mg L^−1^ NO_3_^−^) and pot volume (V1 = 400 cm^3^, V2 = 3200 cm^3^). Results are means ± SD (n = 9). Different letters mean significant differences between the groups, according to Tukey posthoc test (*P* < 0.05). *DAS* days after sowing.

The treatments’ effect on the total chlorophyll content is shown in Fig. 4. At 50, 57 and 64 DAT in 3 out 4 associations between PAW concentration and the volume of substrate, the chlorophyll contents (CCI) in the leaves were almost the same as in the corresponding controls, except the treatment with PAW II in the bigger volume of substrate (V2), for which the CCI was significant higher compared to the control (C V2). For instance, there was a 10.71% increase in chlorophyll content in the PAW II V2 plants compared to that in the C V2 at 64 DAT. The PAW I treatment did not influence in a significant way the chlorophyll content of lettuce plants, regardless of the volume of the pots and the number of days after transplanting. The higher value of chlorophyll measured in the plants treated with PAW II was due to the higher content of NO_3_^−^, which apart from being the main source of nitrogen for plants, it is very important in the chlorophyll formation^23,49^. By having a higher chlorophyll content, the lettuce treated with PAW II have a better quality than the lettuce treated with PAW I or the untreated control. For leafy green vegetables, the chlorophyll content represents a very important quality indicator, because by degradation, the color of the leaves changes leading to a loss of product quality^49^.

Given the fact that the chlorophyll content of PAW II plants is higher compared to the rest of the treatments, we can assume that for the plants treated with 3.0 mg L^−1^NO_3_^−^ the photosynthetic activity might be more intense. In general, an increase in the chlorophyll content is associated with an increased rate of photosynthesis and total plant metabolism^50^. Similarly, Kučerová et al*.*^43^ recorded an increase of 17% in the chlorophyll content of wheat after the plants were irrigated with PAW activated 2 min mL^−1^. Also, a positive effect of PAW treatment on the chlorophyll content was observed by Manniruzzaman et al.^51^.

In our research, the higher PAW concentration (3.0 mg L^−1^ NO_3_^−^) had definitely a positive effect on the chlorophyll content of lettuce plants when grown in pots with the volume of 3200 cm^3^,compared to the corresponding untreated plants. This suggests that PAW II exhibits a better long-term effect than a short-term effect on the seedling’s growth.

### Influence of PAW on biometrical indicators

Leafy green vegetables are usually exposed to the intense action of biotic and abiotic factors because the harvest has to be done in a short period of time^52^.

#### Effect of PAW treatments on the root weight of lettuce plants

The data regarding root weight of lettuce plants corresponding to PAW treatments and pot volume are shown in Table 1. The PAW treatments did not influence the root weight of lettuce plants regardless of the concentration used (1.5 mg L^−1^ NO_3_^−^ or 3.0 mg L^−1^ NO_3_^−^), the day of determination (50, 57 or 64 DAT), or the pot volume (V1 = 400 cm^3^ and V2 = 3200 cm^3^). The differences between the root weights of treated plants compared to controls were not statistically significant. However, the values registered for the groups treated with PAW were higher than those registered for controls. For instance, an increase of 15.71% and 2.30% was registered for PAW I V1 and PAW I V2 and of 21.58% and 3.74% for PAW II V1 and PAW II V2 compared to controls; higher differences being observed for PAW II treatment. Also, it was observed that the plants grown in bigger pots (V2) had a higher root weight for all the groups, treated or not treated with PAW. Among PAW treatments, regardless the pot volume, even if the differences were not significant, PAW II showed a higher plant root weight compared to PAW I, suggesting that a higher concentration of reactive species is more favorable for root development.

**Table 1:** Root weight of lettuce plants grown under different plasma activated water (PAW I and PAW II) treatment and pot volume (V1 and V2) at 50, 57 and 64 DAT.

Treatment	Pot volume	Plant root weight (g)		
		50 DAT	57 DAT	64 DAT
Control	V1	3.95 ± 0.17a	8.09 ± 1.37abcdef	9.36 ± 1.77bcdef
	V2	4.75 ± 0.22ab	9.66 ± 0.21cdef	11.76 ± 1.41f
PAW I	V1	4.02 ± 0.5ab	11.09 ± 0.78ef	10.83 ± 0.55def
	V2	5.61 ± 0.06abcd	11.12 ± 0.7ef	12.03 ± 0.74f
PAW II	V1	5.33 ± 0.77abc	11.35 ± 0.97f	11.38 ± 0.42f
	V2	5.74 ± 0.41abcde	11.87 ± 0.87f	12.2 ± 0.85f

Data are presented as mean ± SD (n = 3). Different letters mean significant differences between the groups, according to Tukey posthoc test (*P* < 0.05).

*DAT* days after transplanting.

#### Effect of PAW treatments on the weight of the lettuce plants

The yield of lettuce is in a great measure correlated with the biometrical indicators, especially with the total weight of leaves and the leaf area.

The weight of the lettuce plants at 50, 57 and 64 DAT is shown in Table 2. At 50 DAT the plant foliar weight was higher for the lettuce grown in pots of 3200 cm^3^ volume (V2) compared to those grown in pots of 400 cm^3^ volume (V1), with values between 89.74% for PAW II group and 150.77% for PAW I. However, the lowest values were recorded for PAW I, but the differences between the lettuce plants from the small pots were not statistically significant. For V2, the differences compared to the control were between 5.31 and 31.80%, which also indicates that the NTP treatment is effective after 50 DAT.

**Table 2:** Plant foliar weight of lettuce plants grown under different plasma activated water (PAW I and PAW II) treatments and different pot volumes (V1 and V2) at 50, 57 and 64 DAT.

Treatment	Pot volume	Plant foliar weight (g)		
		50 DAT	57 DAT	64 DAT
Control	V_1_	14.91 ± 0.58ab	22.96 ± 0.99abc	27.2 ± 0.78bcd
	V_2_	29.38 ± 0.7cd	49.41 ± 0.78ef	75.11 ± 3.26g
PAW I	V_1_	12.34 ± 1.16a	20.06 ± 1.88abc	27.38 ± 0.3cbd
	V_2_	30.94 ± 1.19cd	53.59 ± 1.24f	83.23 ± 5.72gh
PAW II	V_1_	20.41 ± 1.36abc	25.01 ± 1.01abc	32.34 ± 1.42cd
	V_2_	38.72 ± 0.81de	59.23 ± 2.07f	93.88 ± 2.76h

Data are presented as mean ± SD (n = 3). Different letters mean significant differences between the groups, according to Tukey posthoc test (*P* < 0.05).

*DAT* days after transplanting.

Similar results were also registered at 57 DAT, when the foliar weight of plants grown in big pots (V2) was higher compared to the weight of plants grown in small pots (V1), the smallest values being recorded for the lettuce treated with PAW I. The variation between the two types of pots, regardless of the treatment applied increased between 115.18% for control and 167.08% for PAW I. The increases for V2 group induced by the treatments varied between 8.44% for PAW I and 19.87% PAW II, compared to the control.

At 64 DAT, the smallest values of the plant foliar weight were registered for control, regardless the volume of the pot used, compared to PAW I and II where the increases were up to 18.88% for V1 and 24.99% for V2 (both for PAW II treatment).

Between the pot types, the differences varied between 176.13% for the control and 203.96% for PAW I. The higher plant foliar weight for V2 group compared to V1 group, regardless the treatment, was due to the bigger pot volume which allowed a better radicular development, even if the differences were not significant compared to the control (Table 1), enhancing the nutrient uptake and therefore improving the foliar growth. It is well established that a plant with a better root system leads to a larger amount of leaves^49^.

Among PAW treatments, the lettuce treated with PAW II gave a higher plant foliar weight than treated with PAW I, confirming the superiority of the long-term effect of the higher PAW concentration. This result can be attributed to the more important NO_3_^−^ content of PAW II.

#### Effect of PAW treatments on the foliar area of lettuce

The leaf area of lettuce is presented in Table 3. The PAW II treatments at 57 and 64 DAT significantly influenced the foliar area surface of lettuce plants grown in high volume pots (V2); for instance at 64 DAT it was registered an approximately 17.17% increase in the foliar area of PAW II V2 plants compared to the V2 control In addition, it was observed that for all the plants grown in big pots (V2) the foliar area was significant higher compared to lettuce grown in small pots (V1), the differences being 162.5% for control, 176.15% for PAW I and 150.43% for PAW II. The smallest values of the foliar area were registered for the untreated plants regardless of the pot volume used and the day of determination. Among PAW treatments, no significant differences were registered for the same pot volume. Larger surfaces were registered for PAW II treated plants compared to PAW I treated (and both are more important than the control’s), indicating the importance of a higher content of nitrogen.

**Table 3:** The foliar area of lettuce grown under different plasma activated water (PAW I and PAW II) treatments and pot volumes (V1 and V2) at 50, 57 and 64 DAT.

Treatment	Pot volume	Foliar area (cm^2^)		
		50 DAT	57 DAT	64 DAT
Control	V1	489.95 ± 29.49a	817.48 ± 37.72abcd	859.31 ± 14.95abcd
	V2	871.79 ± 81.53bcd	1606.53 ± 13.85e	2255.73 ± 89.53 fg
PAW I	V1	511.48 ± 8.14ab	732.7 ± 81.52abcd	865.79 ± 30.24abcd
	V2	1041.43 ± 26.29cd	1666.54 ± 33.52e	2390.84 ± 107.16gh
PAW II	V1	681.29 ± 31.24abc	830.18 ± 44.84abcd	1055.38 ± 64.83cd
	V2	1061.47 ± 99.28d	1969.17 ± 6.29ef	2642.95 ± 76.59h

Data are presented as mean ± SD (n = 3). Different letters mean significant differences between the groups, according to Tukey posthoc test (*P* < 0.05).

*DAT* days after transplanting.

#### Effect of PAW treatments on the leaf length of lettuce plants

The leaf’s length of lettuce treated or not treated with PAW grown in two different pot volumes is shown in Table 4. The applied treatments significantly influenced the leaf’s length only at 64 DAT, for the plants treated with 3.0 mg L^−1^ NO_3_^−^ (PAW II) and grown in small pots (V1). There was an approximately 4.29% increase of the leaf length of PAW II V1 plants compared to the V1 control. PAW I treatments did not significantly influence the leaf length regardless the pot volume used, compared to the corresponding control. In addition, no significant differences were noticed between the leaf length of the treated or untreated plants grown in small (V1) and bigger pots (V2). Among PAW treatments, no significant differences were recorded taking into account the pot volume. Similar to the other biometrical indicators analyzed, better results were registered for PAW II treatment.

**Table 4:** The leaf’s length of lettuce grown under different plasma activated water (PAW I and PAW II) treatment and pot volume (V1 and V2) at 50, 57 and 64 DAT.

Treatment	Pot volume	Leaf length (cm)		
		50 DAT	57 DAT	64 DAT
Control	V1	9.91 ± 0.32a	10.48 ± 0.3abc	10.96 ± 0.33abc
	V2	12.19 ± 0.36a	13.29 ± 0.12abcd	13.84 ± 0.25abcde
PAW I	V1	10.62 ± 0.59ab	10.57 ± 0.18abcd	11.03 ± 0.32abcd
	V2	12.09 ± 0.16abcd	13.64 ± 0.6abcde	14.55 ± 0.09bcde
PAW II	V1	11.18 ± 0.32abcd	10.96 ± 0.27abcde	11.43 ± 0.29de
	V2	12.6 ± 0.4abcde	14.21 ± 0.57cde	15.65 ± 0.28e

Data are presented as mean ± SD (n = 3). Different letters mean significant differences between the groups, according to Tukey posthoc test (*P* < 0.05).

*DAT* days after transplanting.

#### Effect of PAW treatments on the leaf width of lettuce plants

The results of PAW treatments on the leaf width of lettuce are presented in Table 5. The PAW I or II treatments did not influence in a significant way the width of the lettuce plants, regardless of the volume of the pots used and the days after transplanting. In addition, it was observed that at 50 and 57 DAT there were no significant differences between the leaf width of the treated or untreated plants grown in small and big pots. At 64 DAT for all the plants grown in big pots (V2), the leaf width was significant higher compared to those grown in small pots (V1), the differences varying between 32.15% for control, 35.86% for PAW I and 38.71% for PAW II. Among PAW treatments, PAW II determined better results than PAW I, regardless the pot volume, even if not in a significant way.

**Table 5:** The leaf’s width of lettuce plants grown under different plasma activated water (PAW I and PAW II) treatments and pot volumes (V1 and V2) at 50, 57 and 64 DAT.

Treatment	Pot volume	Leaf width (mm)		
		50 DAT	57 DAT	64 DAT
Control	V1	4.19 ± 0.1a	4.57 ± 0.09abcd	4.51 ± 0.1abc
	V2	4.32 ± 0.18ab	5.47 ± 0.1bcdefg	5.96 ± 0.1 fg
PAW I	V1	4.52 ± 0.07abc	4.23 ± 0.22a	4.35 ± 0.16abc
	V2	4.85 ± 0.07abcdef	5.48 ± 0.21cdefg	5.91 ± 0.22efg
PAW II	V1	4.75 ± 0.07abcd	4.31 ± 0.26a	4.65 ± 0.25abcd
	V2	4.77 ± 0.32abcde	5.68 ± 0.21defg	6.45 ± 0.17g

Data are presented as mean ± SD (n = 3). Different letters mean significant differences between the groups, according to Tukey posthoc test (*P* < 0.05).

*DAT* days after transplanting.

Positive effects of PAW treatments on the growth of the plants, other than lettuce, were reported on strawberry, spinach and radish, for the height of plants and the leaf size. The authors have stated that the main reason of growth promotion was the nitrate nitrogen from PAW which entered the plants through roots^53^. Kučerová et al*.* and Maniruzzaman et al. registered incresed fresh weight values for the wheat treated with PAW^43,51^. Park et al., after studying the effect of PAW on different plant species, observed significant increases of the root and stem length of alfalfa, pole beans and watermelons. The formation of nitrates and nitrites in PAW, along with the formation of hydrogen peroxide were considered to be responsible for the growth of the plants^54^. Lindsay et al. observed an increase in the height of radish, marigold and tomato plants. The same authors reported higher shoot masses for the plants grown in PAW^55^. Increases in the length of *Brassica rapa* var. *perviridis* were recorded by Takaki et al. The authors have concluded that nitrate and nitrites from PAW worked as fertilizers, enhancing the growth of the plants^56^.

The statistical correlations among the analyzed morphological and physiological parameters are shown in Table 6. The data indicates in a percentage of approximately 99.85%, a positive correlation between the foliar area and the leaf weight. In addition, a significantly positive correlation was found between the leaf length and width and the foliar area (approximately 99%). Regarding the analyzed morphological and physiological parameters, no significant positive correlation was found between them. For instance, the correlations between the foliar parameters and the photosynthetic pigments are relatively low (51–58%) compared to the root system which is higher (72.5%), suggesting that its development might be due to the accumulation of reserve substances resulted from photosynthesis. Similarly, the results obtained by Caruso et al., for the pepper culture, grown in both organic and conventional systems, showed that in the *Solanaceae* species there is no positive correlation between the content of photosynthetic pigments and the yield, but only between the photosynthesis activity and the yield^57^.

**Table 6:** Statistical correlation coefficients among leaf weight, photosynthetic pigments, root weight, foliar area , leaf length and leaf width of lettuce plants under different plasma activated water (PAW I and PAW II) treatments and pot volumes (V1 and V2).

Parameters	Leaf weight	Pigments	Root weight	Foliar area	Leaf length	Leaf width
Leaf weight	1					
Pigments	0.520632	1				
Root weight	0.804503	0.72592	1			
Foliar area	0.998565	0.511192	0.813881	1		
Leaf length	0.99526	0.581778	0.810238	0.990233	1	
Leaf width	0.992356	0.520948	0.783444	0.993548	0.988291	1

In conclusion, PAW treatments had a significant positive effect on the lettuce foliar weight and area when it was used in a concentration of 3.0 mg L^−1^ NO_3_^−^ and when the pot volume was 3200 cm^3^.

### Effect of PAW on the dry weight of lettuce leaves

The percent of dry weight was more important in the small pots (V1) than in the bigger ones (V2) in case of control and both treatments at all three harvest dates (Table 7). This fact can be explained by faster water evaporation from the soil, due to the small volume of substrate, while the bigger substrate volume is retaining the water for a longer time period and is providing it gradually to the plant, and thus the moisture of lettuce grown in bigger pot is more important. The PAW treatments stimulate the mass accumulation in case of 50 DAT in both types of pots, while at 57 DAT—only in big pots and at 64 DAT—only in small pots. This mass accumulation could be explained by a stimulation of protein synthesis, induced by PAW. The positive effects of PAW treatments on the dry weight of plants were also reported by Ling et al*.* who registered increases in the shoot and root dry weight (21.95%, and 27.51%, respectively) of soybean compared to the untreated control^58^.

**Table 7:** The dry weight of lettuce plants grown under different plasma activated water (PAW I and PAW II) treatment and pot volume (V1 and V2) at 50, 57 and 64 DAT.

Treatment	Pot volume	Dry weight (%)		
		50 DAT	57 DAT	64 DAT
Control	V1	6.91 ± 0.0289gh	7.737 ± 0.0819j	6.887 ± 0.0294g
	V2	5.69 ± 0.0288e	4.713 ± 0.0295a	5.407 ± 0.0293d
PAW I	V1	7.727 ± 0.0292j	7.077 ± 0.0297hi	8.317 ± 0.0297 k
	V2	5.987 ± 0.0291f	5.123 ± 0.0296c	4.8 ± 0.0289ab
PAW II	V1	7.00 ± 0.0306ghi	7.133 ± 0.0333i	7.767 ± 0.0296j
	V2	6.097 ± 0.0296f	5.197 ± 0.0291c	4.933 ± 0.0333b

Data are presented as mean ± SD (n = 3). Different letters mean significant differences between the groups, according to Tukey posthoc test (*P* < 0.05).

*DAT* days after transplanting.

### Effect of PAW on the nitrites and nitrates content in lettuce leaves

Nitrites and nitrates are the predominant sources of nitrogen absorbed by the plants for their growth, from the soil solution in the soil crop, or from nutritive solution in hydroponic crops. PAW generation can be an alternative source of nitrogen for plant growth.

The nitrites content was very small for all studied samples, being between 0.004 mg NaNO_2_/g of dry weight (DW) (for control in big pot at 57 DAT) and 0.02 mg NaNO_2_/g DW (for PAW I treatment in small pot at 64 DAT) (Table 8). It was noticed that generally, the lettuce grown in big pot (V2) had lower nitrites content than the lettuce grown in small pot (V1), but not in all cases the differences are statistically significant. Contrary, the nitrates content (Table 9) was much more important (more than × 100 times) in the lettuce grown in big pots (up to 36.4 mg KNO_3_/g DW), compared to small pots (under 0.3 mg KNO_3_/g DW).

**Table 8:** The nitrites content of lettuce plants grown under different plasma activated water (PAW I and PAW II) treatment and pot volume (V1 and V2) at 50, 57 and 64 DAT.

Treatment	Pot volume	Nitrites content (mg NaNO_2_/g DW)		
		50 DAT	57 DAT	64 DAT
Control	V1	0.005 ± 0.0001b	0.013 ± 0.0001e	0.01 ± 0.0001d
	V2	0.005 ± 0.0003b	0.004 ± 0.0001a	0.005 ± 0.0001b
PAW I	V1	0.010 ± 0.0001cd	0.014 ± 0.0002f	0.02 ± 0.0003g
	V2	0.009 ± 0.0003c	0.005 ± 0.0001b	0.005 ± 0.0003bd
PAW II	V1	0.012 ± 0.0002e	0.012 ± 0.0005e	0.01 ± 0.0001cd
	V2	0.006 ± 0.0003b	0.006 ± 0.0002b	0.005 ± 0.0001b

Data are presented as mean ± SD (n = 3). Different letters mean significant differences between the groups, according to Tukey posthoc test (*P* < 0.05).

*DAT* days after transplanting.

**Table 9:** The nitrates content of lettuce plants grown under different plasma activated water (PAW I and PAW II) treatments and pot volumes (V1 and V2) at 50, 57 and 64 DAT.

Treatment	Pot volume	Nitrates content (mg KNO_3_/g DW)		
		50 DAT	57 DAT	64 DAT
Control	V1	0.238 ± 0.0095a	0.098 ± 0.0275a	0.108 ± 0.0176a
	V2	17.626 ± 1.7759b	35.178 ± 3.4118c	23.454 ± 1.5028b
PAW I	V1	0.162 ± 0.0398a	0.061 ± 0.0174a	0.271 ± 0.0127a
	V2	16.417 ± 0.9963b	22.639 ± 1.7632b	36.406 ± 4.903c
PAW II	V1	0.119 ± 0.0388a	0.033 ± 0.0107a	0.076 ± 0.0209a
	V2	17.107 ± 0.7524b	22.318 ± 2.5788b	35.603 ± 5.7657c

Data are presented as mean ± SD (n = 3). Different letters mean significant differences between the groups, according to Tukey posthoc test (*P* < 0.05).

*DAT* days after transplanting.

The PAW treatment generates oxidative stress in plants, as a consequence of the contained active species as H_2_O_2_, NO_3_^–^^22,40^. The oxidative stress activates the plant’s antioxidant defense systems, which disrupts the lettuce’s metabolism^59^. Concerning the nitrates content in control samples (Table 9), we noticed an irregular variation due to the harvest date (number of days after transplanting), the maximum value being registered at the second harvest (57 DAT) of the lettuce grown in big pots. Pinto et al*.* stated that it was previously proved that NO_3_^−^ accumulation in plants is the consequence of different factors: time of the year and harvest date, soil NO_3_^−^ phytoavailability, nitratereductase activity, temperature, light intensity or water availability^60^. In a study regarding potatoes it was found that the delay of harvest date decreased nitrate contents in winter–spring crop and increased it in summer–autumn crop^61^.

The PAW treatment slowed the increase of nitrates concentration at the second harvest, probably due to the use of nitrogen for the synthesis of some antioxidants, as a defense reaction to the induced oxidative stress^59^. The PAW treated lettuce from the last harvest (64 DAT) contained much bigger nitrates content than the control, and a possible explanation is that the plant accumulates nitrates from PAW, as a consequence of the general slowdown of plant’s metabolism when plant ages.

Considering the results, we recommend in the case of PAW treated lettuce, the harvest to be done after 57 DAT, as the nitrates content is less than in untreated lettuce, or after 50 DAT, when was registered the smallest nitrate content (in V2).

## Materials and methods

### Lettuce seed sample

Untreated lettuce seeds (Shangore F1) were purchased from Syngenta Ltd. Company. The number of seeds per gram was 1050, the purity 100% and the water content 9%. Before to use, the seeds were kept in a refrigerator at 0–4 °C.

### Plasma activated water technology

The plasma activated water used in this experiment was obtained in a spray water plasma reactor. The technical function of this reactor consist in the directly exposure in the plasma zone of the electrical discharge of the water to be treated. To accomplish this goal the water was introduced in the reactor’s room by the pump (P), under the action of air flow, through the electrode E1 which is also the nozzle. The pump (P) allows the water flow rate to be adjusted, while the air flow rate is adjusted with a rotameter.

**Figure 5:**
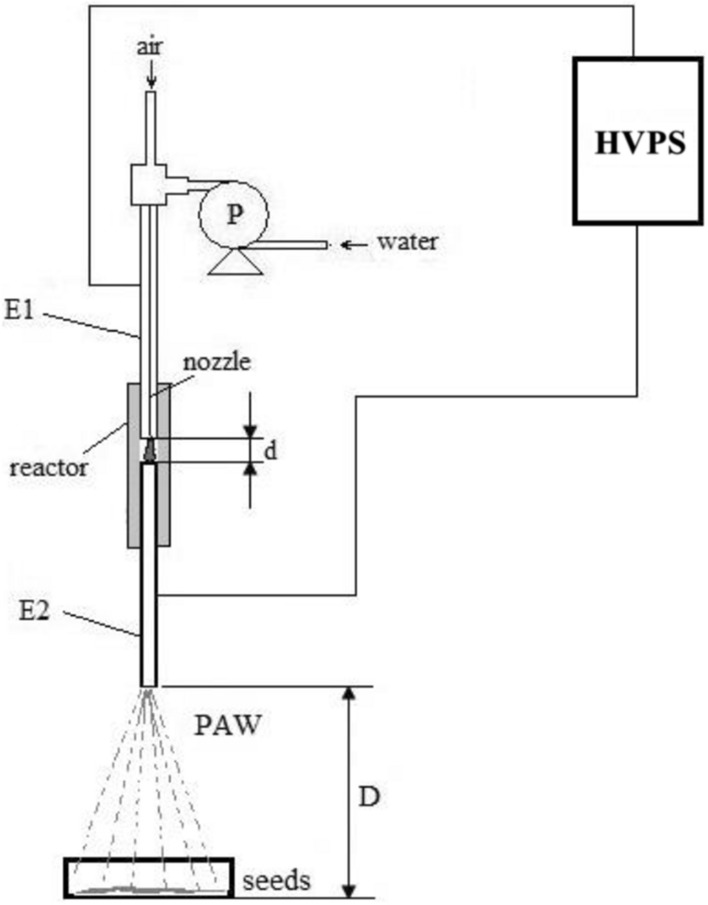
Schematic design of PAW system for seeds treatment. *P* pump; *E1, E2* electrodes; *HVPS* high pulse voltage supply.

The experimental set-up is presented in Fig. 5. The reactor is integrated in an electrical circuit with a high pulse voltage supply (HPVS).

In this configuration of the non-thermal plasma reactor, the electrodes are also elements in the gas and liquid circuits. The electrode E1 functions as an inlet nozzle, while the electrode E2 is the outlet for the PAW. The electrodes are made by INOX, have a tubular design with a 1 mm diameter inside, and are in coaxial position, with 4 mm distance between them, in a glass tube (3 mm diameter inside) as chamber of reaction (Fig. 5).

The mixture of air and distillated water get in the reactor chamber by the inlet nozzle (E1), pass through the active plasma zone, and the mixture get out from the reactor through the outlet tube (E2). The air is released in the environment, and the PAW is applied directly to the seeds of lettuce.

The same electrical parameters were maintained in two different procedures, the air flow was constantly 1.5 L^−1^ min, but the water flow rate was changed. Two different PAW samples, PAW I, respectively PAW II were obtained and their reactive species concentrations are presented in Table 10.

**Table 10:** Treatment application design.

PAW sample	pH	NO_3_^−^ content (mg L^−1^)	H_2_O_2_ content (mg L^−1^)	Debit (l h^−1^)	Applied time (s)
PAW I	6.54	1.5	0.5	3.00	6
PAW II	5.88	3.0	1.65	1.50	12

### Seed germination assay and PAW treatments

Shangore F1 seeds were soaked in distilled water for 5 h, disinfected in 70% alcohol for 2 min and washed thoroughly with sterile distilled water for alcohol removal. The germination test was conducted in Petri dishes, on filter paper of 78 g m^−1^. The seeds were divided into three groups: control—watered with distilled water (pH = 6.9), PAW I and PAW II. Each group was represented by three repetitions, with 100 seeds/repetition.

The seeds were germinated under controlled conditions, in a plant growth chamber (Sanyo MLR 351H), at 20 °C, 80% relative humidity and 1500 lx. It is known that the optimum germination of *Asteraceae* seeds takes place in the light.

During the first 48 h, all the groups were watered with 5 mL distilled water. During this time, the Petri-dishes were kept covered to avoid the water evaporation.

After 48 h, PAW I or II were sprayed directly on the lettuce seeds, in a single-dose treatment, by placing the Petri dishes under the outlet electrode, at a distance of 10 cm. For each Petri dish with 100 seeds, a volume of 5 mL of PAW I or PAW II was applied for 6, respectively, 12 s (s). The control group was treated with the same amount, but using distilled water instead of PAW. After the treatment, in order to keep the moisture, the Petri dishes were watered with distilled water.

The evaluation of seeds’ germination was done in dynamic, starting with the 3rd day after sowing (DAS) until the same value (number of germinated seeds) was obtained in 2 consecutive days, that is the 7th day. The seeds were considered to be germinated when the radicle was at least 1 mm in size.

The germination rate (Rg) was defined as the report between the number of germinated seeds (Ng) and the total number of seeds (Nt) multiplied by 100:

$$Rg=\frac{Ng}{Nt}\times 100$$

### Plant replication and transfer in the greenhouse

At 10 DAS, the seedlings were replicated for rooting in a tray with 288 alveolus, using Kekkilla peat as substrate with the following characteristics: 90% organic matter (OM), 65% moisture; pH 5.9; EC 44 mS m^−1^, soluble nitrogen (N) 1250 ppm; soluble phosphorus (P_2_O_5_) 400 ppm, soluble potassium (K_2_O) 2000 ppm. The seedlings were kept in the same conditions as the seeds, except the light regime whose intensity was 3000 lx during the day and 1000 lx during the night, 12 h/12 h.

In the 14th day after replication, the lettuce plantlets were transplanted in pots of 400 cm^3^ (V1) and 3200 cm^3^ (V2), respectively, using the same substrate to which were added 5 g of organic fertilizer, namely Orgevit with the following composition: total N_2_ 4,0%; P_2_O_5_ 3.0%; K_2_O 2.5%; MgO 1.0%; OM 65%; pH 7. The pots were then transferred in the greenhouse of the University of Agricultural Sciences and Veterinary Medicine (UASVM) of Iasi, Romania, under controlled conditions: 13–14 °C, 70–75% RH, 25,000 lx, during the day and 9–11 °C, 60–65% RH, 1000–2500 lx, during the night. The irrigation was done according to the technological norms available for organic crops grown on artificial substrates^31,33^, by using the Priva fertirigation system. The control of pests and pathogens was carried out in accordance with the literature^62^. The experiment was done in triplicate, each repetition consisting in 15 plants (5 plants for each harvest point). The effects of PAW treatments on several physiological and biochemical parameters, also on yield were registered in dynamic at 50, 57 and 64 days after transplanting (DAT).

### Radicle and hypocotyl length measurements

Twenty seedlings from each Petri dish were randomly selected to measure in dynamic the radicle and the hypocotyl length, starting with the 3rd DAS for radicles and the 5th DAS for hypocotyls.

### The surface area, the length and width of the leaves

These parameters were determined in dynamic at the established time period (50 DAT, 57 DAT, 64 DAT) by using the Li-3100C Area Meter, for all the leaves of 5 plants for each repetition.

### Photosynthetic pigments determination

The chlorophyll content was registered in dynamic at 50, 57 and 64 days after transplanting (DAT) just before the harvest of the lettuce plants. The measurement was done by using the CCM-200 plus Opti-Sciences Chlorophyll Content Meter.

### Roots and leaves weight measurements

The total weight of the roots and leaves was evaluated by using the analytical balance with three decimals (Kern).

### Nitrite and nitrate content

The nitrite and nitrate content determination was done in dynamic at 50, 57 and 64 days after transplanting, for PAW I and PAW II treated lettuce, compared to control.

The samples were dried in a convection oven (Biobase BOV-T25F) at 80 °C ± 2 °C till constant weight and the dry weight (DW) was expressed as percent. In order to extract the nitrates and nitrites, the dry samples were exactly weighted with 4 decimals. After addition of hot distilled water and saturated borax, the extraction was performed on a shaking bath at 97 °C ± 1 °C for 20 min. After cooling, solutions of potassium hexacyanoferrate (II) and zinc acetate were added. After 30 min, the extracts were filtered using quantitative filter paper and the total volume was brought to 25 mL with distilled water. The nitrite content was determined using Analytik Jena 200 Plus spectrophotometer following Griess method. Results were expressed as mg NaNO_2_/g DW from a calibration curve. The nitrate content was measured after reduction of nitrites to nitrates in presence of cadmium, according to the same procedure^63^, and results were expressed as mg KNO_3_/g DW.

### Statistical analysis

The data are expressed as the means ± standard deviation (SD). The statistical evaluation of the results was carried out by one-way and two-way ANOVA with replication, followed by Tukey’s test with a degree of confidence of 95% (*P* < 0.05), by using SPSS version 21.0 (2012).

## Data Availability

All data generated or analysed during this study are included in this article.
